# The Advantage of Microelectrode Recording When Pneumocephalus Threatens the Precise Placement of a Deep Brain Stimulator

**DOI:** 10.5334/tohm.1098

**Published:** 2025-12-29

**Authors:** Nur Walker-Pizarro, Sara Robledo-Rengifo, A. Enrique Martinez-Nunez, Tejas R. Mehta, Dorian M. Kusyk, Kelly D. Foote, Joshua K. Wong, Michael S. Okun

**Affiliations:** 1 Department of Neurology, Norman Fixel Institute for Neurological Diseases, University of Florida, Gainesville, Florida, United States; 2 Department of Neurology, Colombian Neurological Institute, Medellin, Colombia; 3 Department of Neurosurgery, University of Florida, Gainesville, Florida, United States

**Keywords:** Parkinson’s Disease, Deep Brain Stimulation, Subthalamic nucleus, Microelectrode recording, Pneumocephalus, Brain shift

## Abstract

**Clinical Vignette:**

A 69-year-old woman with Parkinson’s disease underwent left subthalamic nucleus (STN) deep brain stimulation (DBS). Intraoperative awake microelectrode recording (MER) was used to confirm targeting.

**Clinical Dilemma:**

MER and stimulation mapping revealed a short STN segment on the central pass, absent STN activity on the lateral pass, and low thresholds for capsular side effects. The data suggested a mismatch between the planned imaging-based trajectory and the localization of STN using physiology.

**Clinical Solution:**

A substantial adjustment based on MER was required, giving up the ‘fork’ in the brain. The lead was repositioned 3.4 mm posterior and 2.9 mm medial to the initial central pass (4.9 mm vector). Final placement produced robust motor benefit and a desirable therapeutic window for programming.

**Gap in Knowledge:**

Asleep image-guided workflows assume static intracranial anatomy: pneumocephalus can induce millimetric brain shift. This case demonstrated a pneumocephalus-related displacement and how MER, stimulation thresholds, and postoperative atlas-based validation can be employed to correct it.

**Highlights:**

This case illustrates how intraoperative pneumocephalus can compromise targeting in deep brain stimulation.

Microelectrode recording provided critical confirmation, guided corrective lead adjustments, and safeguarded therapeutic outcomes, emphasizing the value of physiology-based targeting alongside modern imaging techniques.

## Clinical Vignette

A 69-year-old woman with a 12-year history of idiopathic Parkinson’s disease and a prior right -sided subthalamic nucleus (STN) deep brain stimulation (DBS) device placed in 2018 was evaluated for a left-sided DBS placement. She presented with significant motor fluctuations, dyskinesias, and medication-refractory tremor despite optimized pharmacological therapy and placement of a unilateral DBS device.

Preoperative targeting was planned by a neurosurgeon using direct visualization of the STN on a T2-weighted brain MRI. The trajectory chosen was planned to pass through the posteromedial STN and was defined as the central pass. Two simultaneous MER passes were used: a central trajectory corresponding to the neurosurgeon’s planned target and a lateral trajectory placed 2 mm away from the central trajectory. The absence of STN firing on the lateral pass suggested the trajectory was located outside the STN. The central MER pass revealed no thalamic activity, a short STN segment (3.8 mm) and low thresholds (1.2–1.3 mA) for capsular side effects during macrostimulation conducted from the guide tube at 3 mm above and again at 5 mm above the intended STN target. These findings together, with absent thalamic activity, suggested that the planned trajectory was likely anterolateral to the optimal STN region. Collectively, these electrophysiologic and stimulation related findings indicated that proceeding with the planned trajectory risked suboptimal benefit and a narrow therapeutic window.

## Clinical Dilemma

Although asleep and image-guided targeting approaches have been increasingly used as an alternative to awake, MER-guided DBS, the sole reliance on imaging may not account for dynamic intraoperative changes to the brain such as cerebrospinal fluid (CSF) loss or pneumocephalus. What makes this case distinctive is the quantification of an imaging-physiology discrepancy (4.9 mm vector) as well as the integration of MER findings, stimulation thresholds, and postoperative atlas-based reconstruction. The data from this case were collected and applied in real time to address the error. This level of quantitative physiologic-anatomic correlation contrast to many available reports that describe brain shift only in a qualitative manner without offering the possibility for a correction.

In this case, intraoperative MER revealed a short STN segment on the central pass and an absent signal on the lateral pass. These findings, combined with low capsular thresholds strongly suggested that the chosen trajectory was anterior and lateral to the intended target. MER confirmed a mismatch between the anatomically planned target and the physiological localization. This raised a critical decision point: whether to proceed with the anatomically planned trajectory or to modify the lead placement based on real-time electrophysiology. Given the magnitude of discrepancy and the physiologic indicator of suboptimal targeting, the surgical team elected to prioritize MER findings over the original imaging-based plan.

In this context, the sole reliance on preoperative imaging introduced the risk of implanting a lead into a suboptimal position with a narrow therapeutic window for outpatient programming.

## Clinical Solution

To address the discordance between MRI-based targeting and intraoperative MER findings, the surgical team relied on physiology-guided adjustments rather than the initial anatomical plan. Both adjustments were performed through the same burr-hole and were accomplished by redirecting the microdrive angle. During intraoperative reassessment, the first adjustment shifted the trajectory 1.4 mm posterior and 1.4 mm medial to reduce proximity to the internal capsule. This move represented the maximal adjustment that could be made without giving up the ‘fork’ in the brain [1], which is a technique the surgeon can employ to limit brain shift which can occur if the guide tube apparatus is removed. This adjustment resulted in improved, however still unacceptably low thresholds (1.5 mA) for activating the internal capsule during intraoperative test stimulation. Persistently low internal capsule thresholds revealed the need for an additional posteromedial correction.

**Figure 1:**
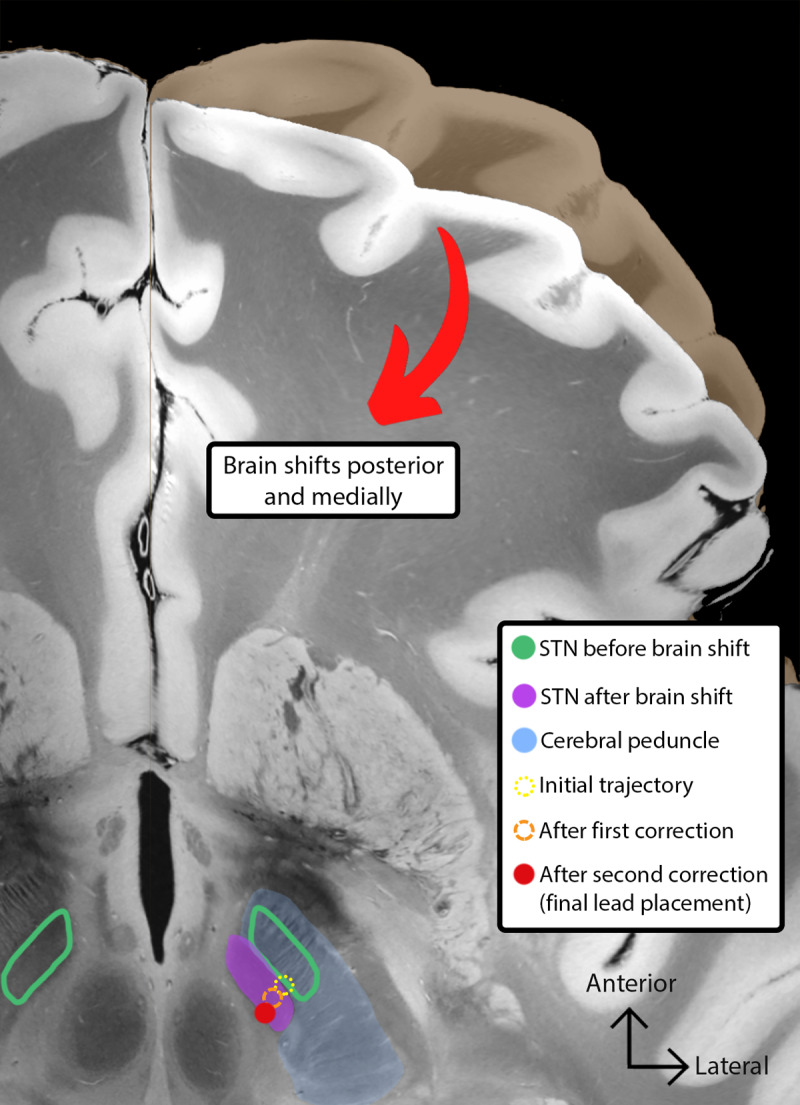
Representation of the subthalamic nucleus (STN) position before and after the brain shift from pneumocephalus, and the trajectories implanted during microelectrode recording (MER), after the first connection, and the final placement. Proximity to the cerebral peduncles leads to lower thresholds for motor side effects. The figure is illustrated on a warped cut of a 7 Tesla ex-vivo MRI section of a human brain. Abbreviations: STN, subthalamic nucleus; MER, microelectrode recording.

A second, larger adjustment required sacrificing the ‘fork’ in the brain by moving an additional 2.0 mm posteriorly and 1.5 mm medially, for a total vector distance of 4.9 mm (Figure 1). This final trajectory produced clear improvement in tremor, rigidity, and bradykinesia, with a maximal benefit at 2.2–2.3 mA and a capsular threshold side effect at 3.5 mA. Immediate post-operative imaging demonstrated significant pneumocephalus. Delayed image-fusion using an atlas-based reconstruction validated the accuracy of the final lead position. This case illustrates an important scenario where awake, physiology-guided DBS uniquely enabled detection and correction of dynamic brain shift. This possibility was realized because the case had access to physiology as a well as imaging and a real-time adjustment could be made to enhance the therapeutic window of the DBS lead that was ultimately placed.

Strategies to mitigate intraoperative brain shift include minimizing dural opening, early dural sealing and burr hole occlusion. All these techniques are designed to limit the loss of cerebrospinal fluid (CSF). Additionally, using a neutral head positioning to reduce the ingress of air has been shown to reduce brain shift [2].

## Gap in Knowledge

Despite extensive discussion of brain shift in DBS surgery, there remains limited guidance on how to objectively identify when intraoperative deviations should necessitate a correction in trajectory. Postoperative CT confirmed the presence of pneumocephalus, and the data strongly suggested brain shift as the cause of the discrepancy. This data corroborated the intraoperative physiology. The *Brainlab Elements* software [Brainlab, Munich, Germany] was used in this case to integrate the preoperative MRI with the intraoperative CT and a brain atlas. This approach was however unable to account for real-time anatomical displacements. The occurrence of brain shift in this case highlights an important potential role of MER during DBS surgery: A safeguard to be used in correcting unintended brain shift [2]. The unique contribution of this case included the triangulation of intraoperative physiology, the quantification of shift magnitude, and the atlas-based reconstruction.

Although imaging-based and asleep DBS workflows have been increasingly adopted, they rely on the assumption that intracranial anatomy will remain static throughout a surgical procedure. This assumption fails in the presence of dynamic intracranial changes such as CSF egress, pneumocephalus, or positioning-related shifts. These complications can and will occur even in experienced centers [2]. In the subthalamic region, where therapeutic margins between optimal motor benefit and internal capsule side effects can be as little as 1–2 mm, even small displacements can convert an apparently accurate trajectory into one that compromises the therapeutic window. Practical intraoperative markers to distinguish acceptable variance from clinically meaningful displacement can be useful to clinicians in an operative setting.

This case quantified a large 4.9 mm posteromedial shift. The MER plus intraoperative stimulation guided a corrective trajectory. MER functioned not only as a secondary check, but as a safeguard for accurate placement under dynamic conditions.

Emerging tools such as deformable registration algorithms and intraoperative MRI have the potential to more accurately model soft tissue dynamics and to integrate electrophysiologic data into real-time navigation platforms [3]. These advances could in the future reduce the reliance on MER. Until such tools mature and become widely available, MER remains a powerful safeguard for detecting and correcting for brain shift during DBS operations.

## Expert Commentary

Beyond illustrating a common intraoperative challenge, this DBS case files introduces a framework for physiologic-anatomic triangulation applicable to a DBS workflow. This case highlights a critical and under-recognized reality of STN-DBS practice: image-guided targeting is vulnerable to dynamic anatomical changes that may unmask following dural opening. Subcortical structures may shift posteriorly during DBS surgery, underscoring the need for dynamic intraoperative verification rather than relying solely on static anatomical assumptions. This shift can be driven by CSF loss, patient age, surgery duration, and can manifest as suboptimal postoperative benefit or unexpected stimulation-induced side effects [2,4,5].

Although image-based and asleep DBS techniques have advanced, they can not always dynamically account for intraoperative brain shift produced by CSF egress, negative-pressure, or air influx [4]. During an awake DBS operation, the atmospheric pressure impacts the relocation of CSF into the spinal compartment, creating a siphon effect that draws extracranial air into the cranial vault, especially when brain atrophy is present [4,6]. These biomechanical forces can produce multi-millimeter displacements without obvious intraoperative signs of change. Such displacements narrow the therapeutic window and can lead to challenges in postoperative programming and management.

In contrast, MER provides a dynamic physiological reference that remains robust even when anatomy moves. This technique offers immediate feedback even when shift is present.

In this case of shift, the MER revealed anterolateral displacement in real time and was useful in guiding a corrective strategy to restore thresholds and clinical benefit. Conventional rigid image-fusion platforms can create a false sense of complete stereotactic accuracy [2]. Postoperative imaging using atlas-based reconstruction can be used to validate the correction and the final lead position.

Single-trajectory corrections through the same burr-hole can be used to minimize tissue passes while preserving therapeutic benefit. Although “keeping the fork” during DBS surgery reduces the risk of brain shift [1], this case demonstrates that substantial displacement can still occur and preserving accuracy sometimes requires sacrificing the ‘fork’ when physiology suggests a misalignment. MER enables clinicians to detect and adapt to such shifts, adjust trajectory, and it provides a safeguard capable of potentially improving therapeutic outcomes. Accurate intraoperative localization reduces the need for complex programming and contributes to a wider and more sustainable therapeutic window. This balance between precision and safety underscores an ongoing need for hybrid strategies to combine the strengths of imaging accuracy with physiological adaptability. In awake DBS, MER not only improves the precision of electrode implantation, but also provides a physiology-based localization of the STN and its surrounding structures. While some studies suggest MER can improve targeting accuracy and clinical outcomes, others have underscored that increasing the number of MER passes may increases the risk of intraoperative hemorrhages [3,7]. Balancing precision with procedural safety remains an active focus for all DBS research.

The ongoing debate between “awake versus asleep” and “MER versus non-MER” approaches should evolve toward adaptive frameworks that can be used to detect and correct intraoperative brain shift in real time. Future advances in intraoperative imaging and deformable atlas registration may complement physiology-based methods, however until such tools emerge, MER can serve as a safeguard against intraoperative brain shift.

Beyond this specific case, this scenario emphasizes a broader principle: intraoperative physiology should not be viewed as a redundant safeguard but as a valuable tool to accompany imaging when millimeter precision can determine clinical outcome. Practical measures to reduce shift include minimizing CSF loss, limiting dural opening, maintaining neutral head positioning, and early sealing of the burr-hole. When MER or stimulation thresholds deviate from expectation, clinicians should consider brain shift.

Comparing imaging-only, physiology-guided, and hybrid approaches in DBS cases complicated by intraoperative brain shift will help us to develop best practices.
